# DuBA.flow—A Low-Cost, Long-Read Amplicon Sequencing Workflow for the Validation of Synthetic DNA Constructs

**DOI:** 10.1021/acssynbio.3c00522

**Published:** 2024-01-31

**Authors:** Adán A. Ramírez Rojas, Cedric K. Brinkmann, Tania S. Köbel, Daniel Schindler

**Affiliations:** † Max Planck Institute for Terrestrial Microbiology, Karl-von-Frisch-Str. 10, 35043 Marburg, Germany; ‡ Center for Synthetic Microbiology, Philipps-University Marburg, Karl-von-Frisch-Str. 14, 35032 Marburg, Germany

**Keywords:** Synthetic biology, long-read sequencing, DNA construct validation, colony PCR, laboratory automation, dual barcode amplicon sequencing

## Abstract

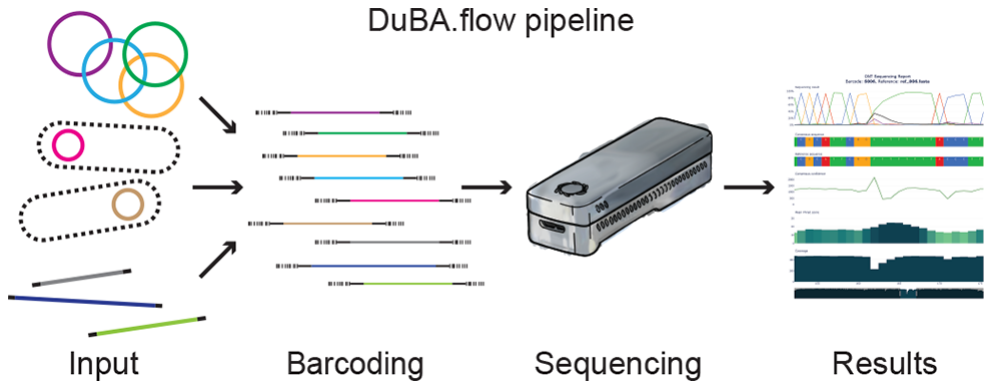

Modern biological science, especially synthetic biology, relies heavily on the construction of DNA elements, often in the form of plasmids. Plasmids are used for a variety of applications, including the expression of proteins for subsequent purification, the expression of heterologous pathways for the production of valuable compounds, and the study of biological functions and mechanisms. For all applications, a critical step after the construction of a plasmid is its sequence validation. The traditional method for sequence determination is Sanger sequencing, which is limited to approximately 1000 bp per reaction. Here, we present a highly scalable in-house method for rapid validation of amplified DNA sequences using long-read Nanopore sequencing. We developed two-step amplicon and transposase strategies to provide maximum flexibility for dual barcode sequencing. We also provide an automated analysis pipeline to quickly and reliably analyze sequencing results and provide easy-to-interpret results for each sample. The user-friendly DuBA.flow start-to-finish pipeline is widely applicable. Furthermore, we show that construct validation using DuBA.flow can be performed by barcoded colony PCR amplicon sequencing, thus accelerating research.

## Introduction

One of the driving forces behind synthetic biology and other molecular biology disciplines is the constant construction and characterization of DNA sequences for downstream applications. Decreasing costs of DNA synthesis and technological advances, particularly in DNA assembly technologies, provide researchers with the necessary cargo and tools.^1^ Researchers are able to design, build, and test large heterologously expressed pathways, e.g., for the production of valuable compounds, or to engineer the organism of choice, e.g., for the valorization of cheap and sustainable raw materials, which is particularly accelerated in environments with laboratory automation.^2,3^ The construction of DNA is a critical part of the build-to-understand approach of synthetic biology, in which researchers refactor or redesign genes, pathways, and entire genomes to understand the fundamentals of life.^4^ All of these efforts are driving the application of biology in the disciplines of biotechnology and biomanufacturing, with the dream of creating a circular bioeconomy that enables the sustainable and economic generation of products demanded by humanity.^5,6^

When DNA constructs are built, a fundamental part of the process is to verify their sequence. Typically, verification is performed in a multistep fashion; an initial diagnostic analysis in the form of a colony PCR or restriction digest is followed by sequence validation by Sanger sequencing. Sanger sequencing is the most widely used commercially available service for a limited number of short sequences in the laboratory routine. However, it becomes impractical and costly as the number of sequencing reactions increases. Recently, methods for performing plasmid sequencing on short-read next generation sequencers in combination with acoustic dispensers have been described to enable economical sequencing of plasmids.^7,8^ Besides the advantages of short-read sequencing in terms of accuracy, its use requires a significant investment in equipment, which can be a barrier to its routine application in laboratories. The Nanopore sequencing platform from Oxford Nanopore Technologies requires little investment, and the quality of sequencing is constantly improving.^9^ As a result, Nanopore sequencing can be adapted to in-house procedures, speeding up workflows and enabling high-throughput, parallelized validation of DNA sequences. Different methods for in-house long-read sequencing workflows have been described as either amplicon-based or transposase-based.^10−12^

Here, we promote DuBA.flow, an in-house dual barcode amplicon sequencing approach for parallelized long-read sequencing for verification of plasmids and amplified DNA. In contrast to Currin and co-workers, we perform a two-step PCR to maximize the reusability of the barcode primers. We developed an accompanying automated analysis pipeline to facilitate fast and reliable data analysis with easy-to-interpret output files. Furthermore, we show that the workflow can be used to generate amplicons for sequencing directly from *Escherichia coli* colonies. In combination with laboratory automation, reaction volumes can be reduced, and highly competitive pricing can be achieved; we sequenced 1536 amplicons on a single Flongle Flow Cell, resulting in an estimated cost of 0.10 € per sample. We further show that the pipeline is compatible with transposase-based fragmentation.

## Results and Discussion

### Dual Barcode Amplicon Workflow for Construct Validation

**Figure 1:**
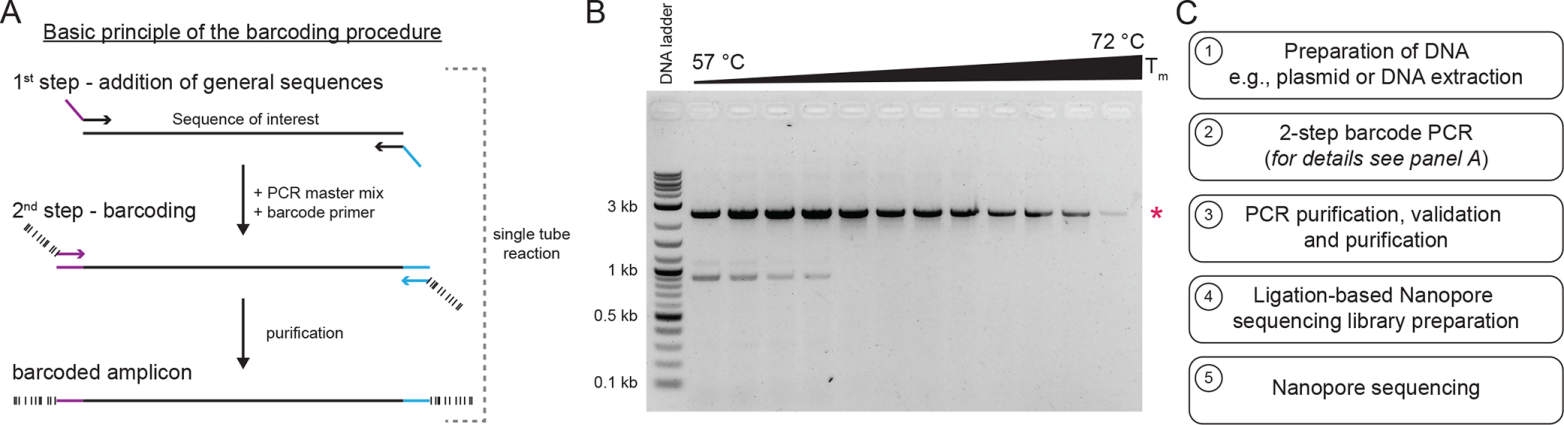
Concept of dual barcode construct validation and its workflow. (A) Basic principle of the dual barcode approach. In a first step, specific primer pairs for a target of interest generate a small fraction of amplicons with general sequences attached (here, M13 forward [purple] and reverse [blue] sequences). Selected forward and reverse barcode primer pairs are added to the same tube with the PCR reaction mix to generate the barcoded target amplicon (see the Materials and Methods section for details). This approach allows reuse of barcode primers by changing only the specific primer pair and provides an economical and versatile solution for in-house long-read amplicon sequencing. (B) New primer pairs for the initial PCR should always be optimized to obtain a single specific band to avoid unspecific amplicons in the second step when the barcodes are attached. This can be done by gradient PCR as shown in the example. With increasing annealing temperatures, unspecific bands disappear. The red asterisk indicates the expected amplicon. (C) The amplicon sequencing workflow starts with the generation of template DNA, followed by the two-step barcode PCR, its validation (e.g., gel electrophoresis), purification, and subsequent Nanopore sequencing library generation and sequencing. Depending on the complexity of the template generation, the workflow can be completed within a single day.

Nanopore sequencing has proven to be a reliable technology for in-house genome and amplicon sequencing. Currin and co-workers described a dual barcode strategy to multiplex large numbers of constructs in a single sequencing experiment. To increase the usability of the barcode primers, we developed a two-step PCR procedure that allows the reuse of the barcode primers and requires only the modification of the amplicon-specific primer pair (Figure 1A). In the first step, amplicon-specific primer pairs anneal to generalized sequences and generate the subsequent PCR template. The barcode primers bind to the attached sequences and are added to the initial PCR reaction to generate barcoded amplicons. After purification, the barcoded sequences can be pooled and used for sequencing library preparation. For traditional reasons, the generalized sequences used in this study are the M13 forward and reverse sequences. The M13 primer sequences may not be compatible with all applications; e.g., the sequences are already present in the template DNA. However, the sequences can be adapted to the user’s needs. The use of 96 forward and reverse barcode primers results in >9000 unique barcoded amplicons for highly multiplexed sequencing experiments. The number of barcodes used can be extended according to the user’s needs.

Importantly, the approach allows reuse of the forward and reverse barcode primer collection by changing only the specific primer pair for the first step, allowing the procedure to be quickly adapted to any type of plasmid and beyond (e.g., validation of genomic integrations and 16S or ITS sequence amplification). A critical step in changing the primer pair is to validate the optimal conditions for the specific primers for subsequent workflow. The annealing temperature and primer concentration must be tested to obtain the maximum yield of barcoded amplicons. An example is shown in Figure 1B, where an unspecific amplicon disappears with increasing annealing temperatures. Unspecific amplicons reduce the data for the target amplicon, because they can serve as a template for the barcode primers in the second PCR. The sequencing pipeline has evolved over time and has been used in many different experiments. Figure 1C provides an overview of the experimental workflow from DNA preparation to sequencing; detailed information is provided in the Materials and Methods section. In general, after amplicon pooling, the ligation sequencing kit is used and libraries are sequenced on Flongle Flow Cells, typically yielding 0.3 to 1.2 GB of sequence data. The raw data obtained is basecalled and can then be analyzed using the developed automated computational pipeline presented in the next section.

### Computational Pipeline with Easy-to-Interpret Sequencing Report

**Figure 2:**
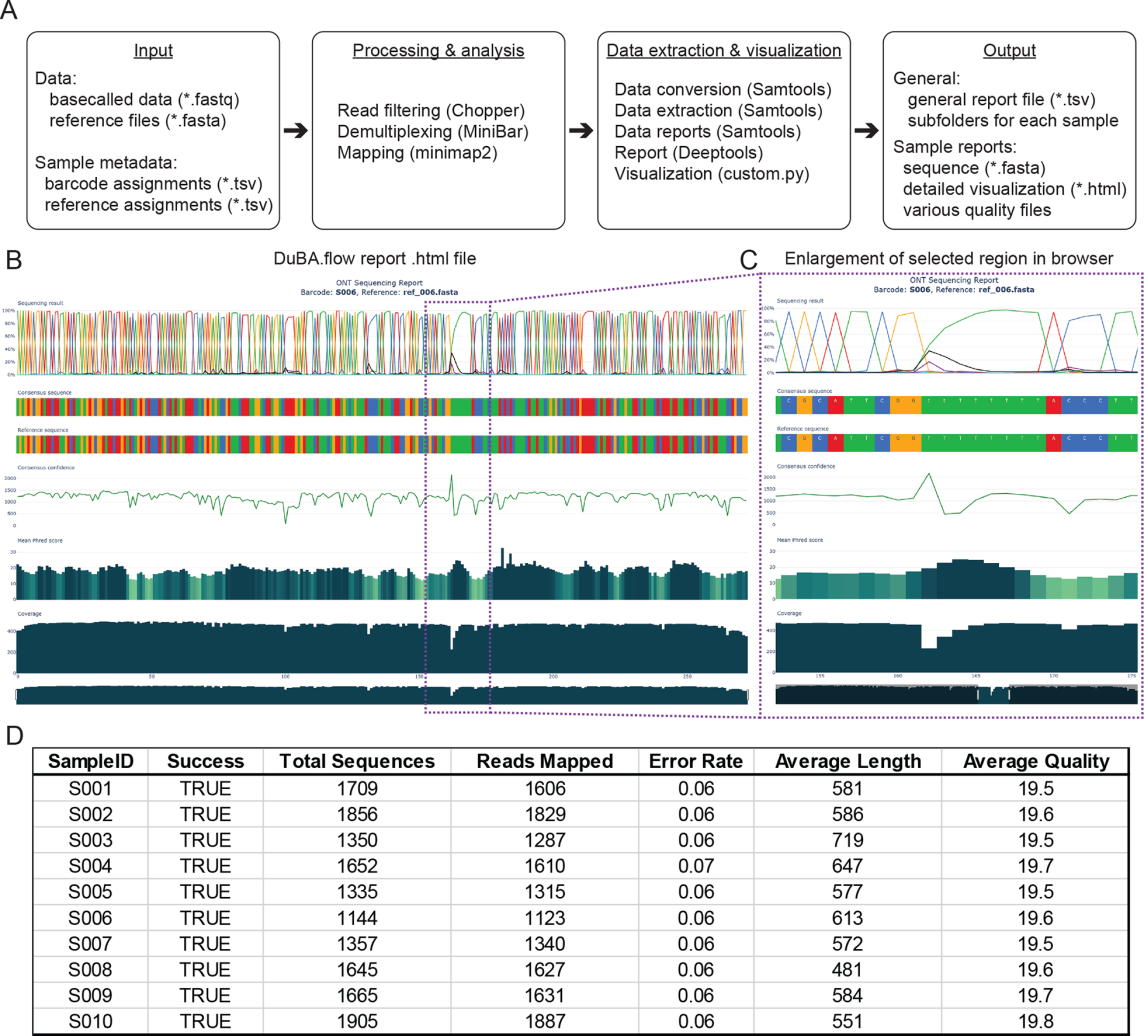
Automated Nanopore sequencing data analysis pipeline of DuBA.flow. (A) Workflow of the automated analysis pipeline. The user has to provide the sequencing and reference data and two sample metadata files. The pipeline automatically processes the data, including read filtering, demultiplexing, read mapping, and subsequent steps for data extraction, conversion, and report generation. The pipeline returns to the user easy-to-interpret files in subfolders for each sample analyzed as well as a general overview report. (B) Example of interactive visualization of sequencing results when a results file is opened with a web browser. Each report file provides the user with eight levels of information. The general report header summarizes information about the sample and its reference. This is followed by the sequencing result, consensus sequence, reference sequence, consensus confidence, mean Phred score, and coverage. These can be analyzed in detail by using the navigation bar. Within the browser, the user can move and zoom along the navigation bar, indicated by the purple dotted box. (C) Enlargement of the data visualized in B, showing a region with a polythymine stretch to illustrate the quality of the data and the easy-to-interpret nature of the result file generated. Polystretches are known for low sequence quality with a decrease in consensus confidence, but the eight thymines are still validated by the amplicon sequencing method. Interactive example result files are provided in Supporting Data S1. (D) General output provides an overview of all samples analyzed. The file provides relevant quality metrics useful for initial analysis of large data sets.

An automated computational analysis pipeline was developed with the goal of being easy to use and providing easy-to-interpret sequencing reports. Therefore, the computational pipeline was built as a Docker image on Docker Hub, and its documentation is available on GitHub (https://github.com/RGSchindler/DuBA.flow). The software package runs on all operating systems supported by Docker and is openly available under a CC BY-NC-SA 4.0 license. The workflow of the pipeline is shown in Figure 2A. The user is required to provide (i) the base-called sequencing data in a single fastq file, (ii) a folder of references as fasta files, (iii) the barcode combinations for each sample in a .tsv file, and (iv) a .tsv file assigning the references to each sample (see Materials and Methods and the DuBA.flow GitHub documentation for details). A sample data set is provided with the software documentation on GitHub. Once started, the automated pipeline first checks that all necessary data are provided and in the correct format before starting the analysis. Next, the basecalled reads are demultiplexed (MiniBar^13^) and mapped (minimap2^14^) against the reference provided for each sample. The mapped data are used to extract the consensus sequence and quality metrics (Samtools^15^), and an easy-to-interpret report file for each sequence is generated (DeepTools^16^) in html format compatible with all tested standard web browsers (Figure 2B,C, Supporting Data S1). In addition to the individual report files, a general file is generated that provides an overview of the sequencing results of the entire analyzed data set (Figure 2D). The analysis pipeline can be run on any standard office computer with a compatible version of Docker and does not require any special hardware specifications. The analysis pipeline is compatible with other sequencing output data as long as the corresponding files are provided and meet the criteria of the programs used (e.g., read length and barcodes are not trimmed, *cf*. DuBA.flow GitHub documentation).

For samples with unknown sequences (e.g., complex libraries, 16S or ITS sequences), we offer an additional software package called ref.creator. ref.creator generates reference files for selected or all samples of a sequencing run. The resulting reference files are passed to the DuBA.flow analysis pipeline and allow the analysis described above. ref.creator is built as a Docker image, and its documentation is maintained on GitHub (https://github.com/RGSchindler/Ref.creator/) under the CC BY-NC-SA 4.0 license. In the case of ref.creator, the user is required to provide (i) the base-called sequencing data in a single fastq file and (ii) the barcode combinations for each sample in a .tsv file (see Materials and Methods and ref.creator GitHub documentation for details). The reference files are provided as {sample_id}.fasta files in the output folder for further use.

### Application of Workflow for Direct Colony PCR Sequencing

**Figure 3:**
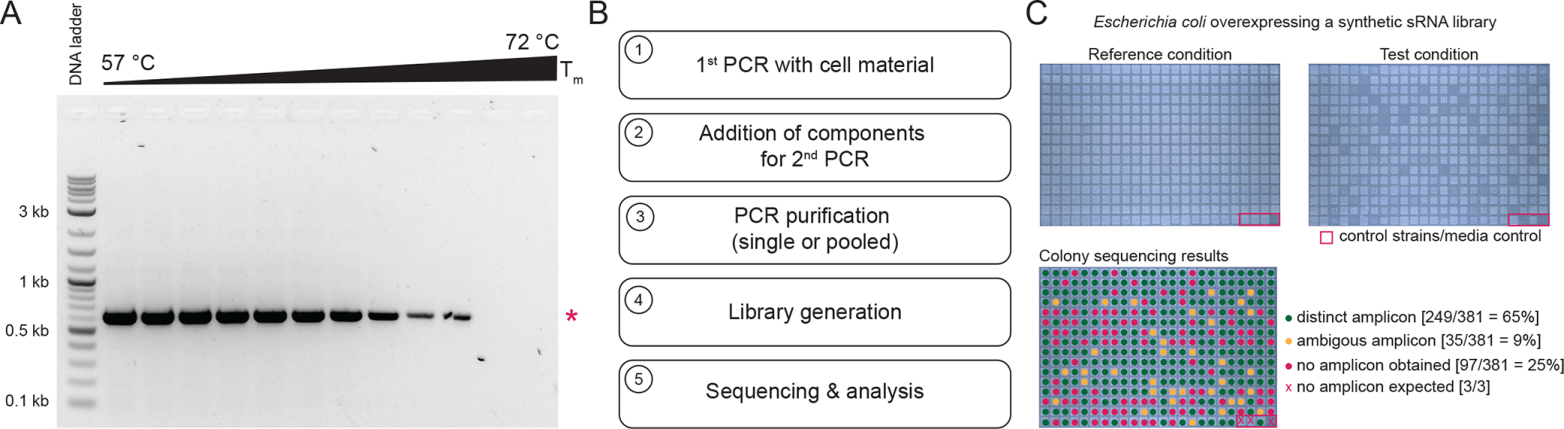
Sequencing of dual barcoded amplicons generated by colony PCR. (A) Determination of the initial PCR conditions by performing a gradient PCR using *E. coli* cells as a template. A representative analysis showing the specificity of the designed primer pair is shown. The black triangle indicates increasing annealing temperatures (*T*_m_). The red asterisk indicates the expected amplicon. (B) Stepwise workflow for colony PCR-based amplicon generation. Steps 1 to 4 can be performed within a normal working day. Data are available for analysis on the second day after overnight sequencing, allowing rapid validation of the DNA constructs in combination with the computational pipeline. (C) Application example of automation assisted colony PCR amplicon sequencing. A library of different DNA constructs was generated by Golden Gate cloning and transformed to *E. coli*. *E. coli* colonies were picked by a colony-picking robot in a 384 grid format and then spotted on a 3 × 3 grid by a screening robot for functional screening (top panels). The reference condition shows all candidates growing (top left), and the test condition (top right) shows candidates with reduced viability. Results and interpretations are described elsewhere. Colonies were used as templates for dual barcode colony PCR and amplicon sequencing success. Approximately 65% of the amplicons validate a specific construct (green dot). Approximately 10% are potentially not individual colonies (orange dot), and approximately 25% did not yield an amplicon (red dot). The red X indicates the control strains and the empty position, where no amplicons were expected. A representative example is shown.

To speed up construct validation and to test workflow limitations, long-read sequencing amplicons were generated by colony PCR (cPCR) from *Escherichia coli* colonies. The described two-step PCR procedure (Figure 1A) was used to amplify the region of interest of plasmids or the genome for subsequent dual barcoded Nanopore sequencing by cPCR from *E. coli* colonies. PCR conditions were optimized for the initial cPCR primer pairs attaching the M13 primer sequences in terms of primer concentration and annealing temperature; this step is recommended for each new primer pair to avoid unspecific amplicons. Figure 3A shows an example in which the cPCR primer pair was tested by gradient PCR to determine the optimal settings for the two-step PCR. The identified optimal settings can then be used to perform the workflow. The workflow is shown in Figure 3B and starts with the initial PCR to add the universal sequences for the barcode PCR. After barcoding, pooling, and amplicon purification, the library is prepared according to the standard procedure using the ligation sequencing kit, followed by sequencing on a Flongle Flow Cell. No differences were observed for amplicons generated from *E. coli* cells compared to amplicons from purified plasmids. The method is further applicable to validate genomic integrations, e.g., genomic integrations in *Saccharomyces cerevisiae* cells were tested. The results suggest that direct amplicon sequencing from microbial colonies and other material is applicable.

### Laboratory Automation Assisted Highly Multiplexed Colony PCR Sequencing

To test the limits of the dual barcoded cPCR sequencing method and to meet the internal demand for construct validation, an automated workflow for the construct validation of transformed *E. coli* cells was designed and validated (Figure 3C). 380 candidate *E. coli* were isolated from each of four different transformed libraries using a colony-picking robot. The libraries were generated by Golden Gate cloning, which combines two basic parts, a PCR-amplified OligoPool library and the destination plasmid (details and results are described elsewhere). The Golden Gate reaction mixture was transformed into *E. coli* MG1655 cells. A colony picking robot was used to pick candidates in a standardized 384-well grid, and after a short incubation the colony material was transferred to 2 μL of initial PCR reaction mix in 384-well PCR plates using a pin-based screening robot. The initial PCR was performed in a thermocycler, followed by the addition of the appropriate barcode primer pair combinations to each well using acoustic dispensers, followed by the addition of the DNA polymerase master mix to a total volume of 5 μL using a contact free nanoliter bulk dispenser. The PCR plate was transferred to a thermocycler for amplification. After the PCR reaction, all four 384-well plates were pooled, purified, and used for Nanopore sequencing library preparation. Sequencing was performed on a single Flongle Flow Cell. In this experiment, individual amplicon purification and DNA measurement was not considered economical; failed candidates could be repeated and added to a subsequent sequencing experiment if necessary. The pooled sample after purification was run on a gel, and the expected smear of DNA fragments ranging from approximately 300 to 600 bp was obtained. After sequencing on a Flongle Flow Cell, >60% of the 1536 amplicon sequences resulted in a specific amplicon for a single candidate. For the remaining sequences, the results were equivocal, with no amplicons, low coverage, or an indication that the isolate may not be a single colony. If necessary, the workflow could be improved. However, it is more economical and less labor intensive to adjust the number of candidates selected based on cloning and sequencing efficiency.

We have sequenced up to 1536 dual barcoded amplicons generated from *E. coli* colonies using our established protocol (see Materials and Methods for details), but this number may not be the limit. The use of direct verification of cPCR amplicons speeds up the experimental workflow, and the low cost would allow, for example, screening four candidates out of 384 transformations in parallel. In the proof-of-concept experiment, the cost is less than 0.10 € per sample, including all steps and consumables. The time from transformation to data receipt can be less than 72 h. On the first day, candidates are isolated, and the two-step PCR protocol is performed. On the second day, the PCRs are pooled and purified, library preparation is performed, and the sequencing run is started. On the third day, the sequencing data were obtained and could be analyzed using the automated computational pipeline.

### Transposase-Based Dual Barcode Approach for Whole Plasmid Validation

**Figure 4:**
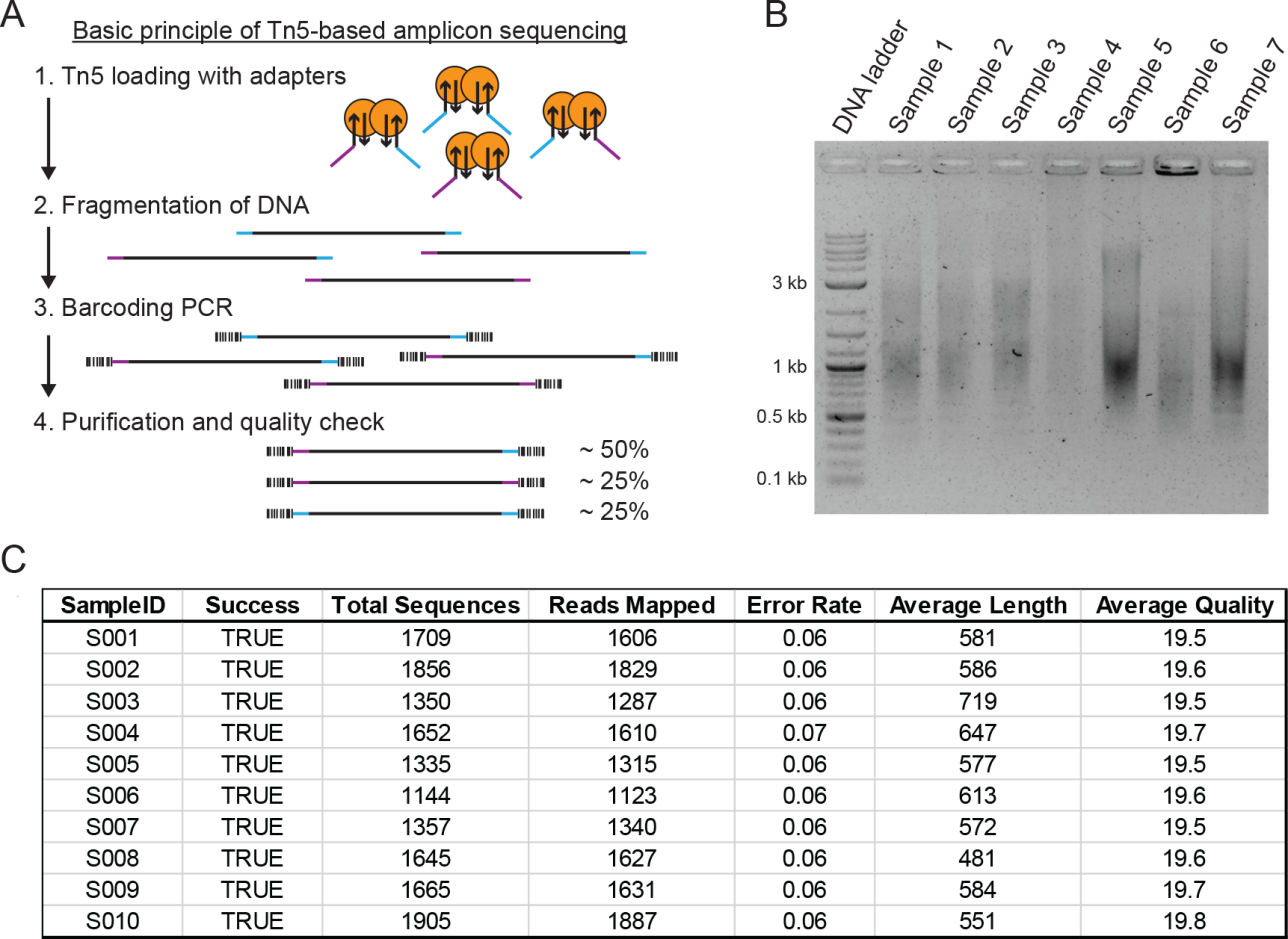
Transposon-mediated dual barcode amplicon sequencing, concept and limitations. (A) Tn5-based library preparation is commonly used to generate short-read next generation sequencing libraries. DNA is fragmented with Tn5 dimers loaded with DNA adapters for subsequent amplification (for details, see Materials and Methods and Henning et al.). The enzymatic fragmentation results in three different types of fragmented DNA. The universal M13 sequences (indicated by purple and blue) were used to reuse the universal barcode primers to generate highly complex dual barcoded sequencing libraries. PCR amplification results in approximately 50% dual-barcoded DNA and 50% mono-barcoded DNA. (B) Example analysis of enzymatically fragmented and amplified DNA (step 4 of panel A) using the Tn5 method of Henning et al. Amplification of the fragmented samples using the barcode primers results in the expected smear on the agarose gel. (C) Data generated by the Tn5 workflow are fully compatible with the automated analysis pipeline. In contrast to amplicon sequencing, the average length is reduced as expected and depends on the enzymatic fragmentation procedure. In the shown sequencing experiment, 200 samples were sequenced, and the results of the first 10 samples are shown as a representative example.

Inspired by the dual multiplexing approach of Currin et al. and the transposase procedure of Henning et al., the dual barcode approach was combined with enzymatic fragmentation using a Tn5 transposase loaded with adapters that provide the binding sequences for the dual barcode primers (Figure 4A). The rationale behind this was to increase the number of multiplexed samples on a single flow cell compared to direct single barcode loading.^10,11^ In addition, this method also allows sequencing of plasmids of unknown sequences, in contrast to the amplicon method. Tn5_R27S,E54K,L372P_ from Henning et al. was purified and used according to the described procedure. Various parameters related to DNA fragmentation were optimized for optimal input for library preparation, resulting in optimal reproducible fragmentation results (Figure 4B; details in Materials and Methods). Multiple sequencing experiments were performed, and adequate data output was obtained (Figure 4C). When sufficient data were obtained for a sample, plasmids could be easily analyzed (Supporting Data S1). However, a disadvantage of this strategy is that a fraction of amplicons contain identical barcodes at both ends (*cf*. Figure 4A). Nevertheless, the method may be relevant in certain workflows, but its limitations must be considered. As an aside, the purified transposase shows stable activity for >18 months when stored at −70 °C.

## Conclusion

We are making available to the community a versatile and economical start-to-end workflow for dual barcode amplicon long-read sequencing, including a dedicated automated computational analysis pipeline for the community. The workflow, called DuBA.flow (**du**al **b**arcode **a**mplicon sequencing work**flow**), uses the Nanopore sequencing platform, which has a low initial investment and laboratory equipment requirements, making it easily accessible to the broad community compared to other sequencing technologies. We show that DuBA.flow works in a manual manner and provide evidence that it is compatible with parallelized and downscaled laboratory automation procedures, allowing us to push the cost per sample below 0.10 €, the limits of which were not explored in this study. We would like to emphasize that previously developed workflows such as those of Currin et al. and Emiliani et al. are very useful; DuBA.flow was designed to address limitations such as the versatility of barcodes for different DNA constructs. In addition, we show that sequencing-ready barcoded amplicons can be generated directly from *E. coli* colonies, speeding up the workflow. We provide a complete computational analysis pipeline with easy-to-interpret output files, making data analysis as easy as analyzing Sanger sequencing traces. DuBA.flow is also compatible with enzymatic fragmentation-based library preparation methods but is limited by reduced data output for dual barcoded amplicons. However, this may become negligible as the performance of flow cells continues to improve. The dual barcode approach could dramatically increase the number of samples analyzed in a single run, allowing the analysis of more samples and eliminating contamination when using a flow cell for multiple runs, as >9000 barcode combinations are available. Nevertheless, we recommend the method of Emiliani and co-workers, which is better suited for in-house whole plasmid sequencing, where in most cases ∼96 barcodes are sufficient. Technology may be moving toward an approach where barcode-free multiplex plasmid sequencing becomes the standard.^17^ However, this would rely on different plasmids from known sources and would not allow for sequencing of multiple candidates of the same construct. It also does not allow for a workflow to identify dedicated isolates of complex libraries. Such a workflow relies on basic knowledge of the sequence for each individual candidate to be sequenced.

Nanopore sequencing is a user-friendly technology that is becoming routine in molecular biology laboratories, and DuBA.flow can be a helpful workflow for the community. In particular, the computational pipeline with its easy-to-interpret reports allows anyone with experience in analyzing Sanger sequencing data to interpret the Nanopore sequencing results.

## Materials and Methods

### Strains and Culture Conditions

Standard laboratory *E. coli* K-12 MG1655 strain derivatives were used in this study. *E. coli* cells were cultured in LB media supplemented with the appropriate antibiotic as needed. In the case of solid growth media, the media were supplemented with 2% agar. Table 1 provides an overview of the relevant strains used in this study.

**Table 1:** Bacterial Strains Used Throughout This Study

name	relevant features	purpose	reference
*E. coli* MG1655	K-12 F^–^ λ^–^	recipient of Golden Gate library transformation for subsequent cPCR amplicon sequencing	(18)
*E. coli* BL21-CodonPlus (DE3)-RIL	F^–^*ompT hsdS*(*rB*^–^*mB*^–^) *dcm*^+^ Tet^R^*gal* λ(DE3) *endA Hte* [*argU ileY leuW* Cam^R^]	overexpression and purification of Tn5_R27S,E54K,L372P_ from Henning et al.	Agilent

### Plasmids and Oligonucleotides Used in This Study

The plasmid pETM11-Sumo3 Tn5 (R27S E54K L372P) was used for overexpression and purification of Tn5_R27S,E54K,L372P_ and was obtained from Henning et al. All other plasmids were used only for testing DuBA.flow and are or will be published elsewhere. Sequence examples are provided in Supporting Data S1. Oligonucleotides were ordered and synthesized from Integrated DNA Technologies in 25 nM or 100 nM scale or as OligoPool. OligoPool contains a library of sequences of varying lengths that are converted to double-stranded DNA using the general library amplification primer pair; details of this method and results are published elsewhere. All oligonucleotides were ordered as standard desalted oligonucleotides either in tubes or in 96-well plates. The standard primers are listed in Table 2, and the barcoded primers are provided in the Supporting Information (Table S1).

**Table 2:** Oligonucleotides Used in This Study

ID	sequence (5′ → 3′)^a^	purpose
SLo0100	5′[phos] CTGTCTCTTATACACATCT	Tn5-ME reverse for transposase loading; Henning et al.
SLo0151	CCCAGTCACGACGTTGTAAAACG	M13 forward primer serving as control primer
SLo0152	AGCGGATAACAATTTCACACAGG	M13 reverse primer serving as control primer
SLo0673	*CCCAGTCACGACGTTGTAAAACG*AGATGTGTATAAGAGACAG	Tn5-ME with added M13 forward sequence
SLo0674	*AGCGGATAACAATTTCACACAGG*AGATGTGTATAAGAGACAG	Tn5-ME with added M13 reverse sequence
SLo1577	*CCCAGTCACGACGTTGTAAAACG*CGTCAATTGTCTGATTCGTTACCA	forward primer for colony PCR amplification with added M13 forward sequence
SLo1578	*AGCGGATAACAATTTCACACAGG*CTTCTCTCATCCGCCAAAACA	reverse primer for colony PCR amplification with added M13 reverse sequence

a Sequence hybridizing is underlined, and attached sequences are in italics.

### Plasmid Transformation

Plasmids were transformed into in-house prepared chemically competent *E. coli* cells using the RbCl method.^19^ Plasmid construction of sequenced constructs is described elsewhere, but generally Golden Gate cloning or Gibson assembly methods were used as described in Köbel et al.

### DNA Extraction and Purification

Plasmid DNA was extracted according to an open source procedure using carboxylated magnetic beads.^20,21^ PCR fragments were purified according to the protocol provided in the same reference, using carboxylated magnetic beads. In all cases, the standard protocols provided (https://bomb.bio/) were used in combination with commercially available magnetic beads (SeraMag Speed Beads, Cytiva, Marlborough, USA).

### General Barcode Workflow

The barcoding workflow relies on two PCR reactions to generate dual barcoded amplicons for multiplexed long-read amplicon sequencing. The first PCR adds standardized overhangs (M13 fwd/rev sequences in this study) that serve as the amplification sequence for the second PCR, which adds the Nanopore sequencing barcodes. The first PCR contains 0.125 μM of each specific amplification primer with M13 fwd/rev sequences in a 5 μL PCR reaction using Q5 DNA polymerase (NEB). PCR settings for the SLo1577 and SLo1578 primer pair: 98 °C for 30 s followed by 10 cycles of 98 °C, 20 s; 66 °C, 20 s; and 72 °C, 30 s with a final extension at 72 °C for 1 min and a hold at 12 °C. It is highly recommended to optimize the conditions for each new primer pair or modified DNA polymerase accordingly (*cf*. Figure 1B). The dual barcodes were attached in the second PCR using 1 μL of 1:10 dilutions of the first PCR as a template in reactions of 7 μL with combinations of the barcode primer pairs (0.2 μM each; Table S1). PCR settings: 98 °C for 30 s followed by 25 cycles of 98 °C, 10 s; 66 °C, 10 s; and 72 °C, 30 s with a final extension time at 72 °C and a hold at 12 °C using Q5 DNA polymerase. All PCR reactions were pooled and purified using an open source magnetic bead purification procedure (see above). DNA concentration was then determined using Nanodrop (ThermoFisher Scientific) and Qubit (Invitrogen) using a broad range and/or high sensitivity assay.

### Acoustic Dispensing of Barcode Primer

The barcode procedure can be parallelized and down-scaled using a combination of an acoustic dispenser (here Echo525 or Echo650T, Labcyte) and a nanoliter bulk dispenser (here, four-channel Cobra, ARI). The initial PCR can be reduced to a total volume of 2 μL by using a contact-free nanoliter bulk dispenser to dispense the PCR master mix (1X Q5 buffer, 0.2 mM dNTPs, 0.125 μM of each primer, and 0.01 U/μL Q5 DNA polymerase). An acoustic dispenser is used to add 25 nL of template DNA to the reaction. The reaction is performed in a 384-well thermocycler with settings as described above. For the subsequent barcoding step, the barcode primers (0.2 μM final concentration) are dispensed into the PCR plate by using an acoustic dispenser that generates user-defined barcode combinations. Next, 3 μL of the second PCR master mix (1X Q5 buffer, 0.2 mM dNTPs, and 0.02 U/μL Q5 DNA polymerase) is added using the bulk dispenser, and the reaction is run in a thermocycler with the same settings as described above. All samples are then pooled, purified, and quality checked by gel electrophoresis prior to library preparation.

### Automation Assisted Colony PCR Procedure

*E. coli* colonies were picked from primary transformation plates in a 384 grid format on solid media on a single well plate (PlusPlate, Singer Instruments) containing the appropriate antibiotic using a colony picking robot (PIXL, Singer Instruments). Prepared plates were incubated at room temperature to 37 °C for several hours or overnight to obtain sufficient cell material. Two microliters of the initial PCR reaction mix containing the general amplification primers (see above for details) was dispensed into a 384-well PCR plate using a contact-free nanoliter dispenser (here, four-channel Cobra, ARI). Cell material was transferred from the agar plate to the 384-well PCR plate by using the Rotor HDA+ screening robot (Singer Instruments). The PCR plate was sealed and transferred to a PCR cycler, and the reaction was run with the following settings: 98 °C for 30 s and 10 cycles of 98 °C, 30 s; 98 °C, 20 s; and 72 °C, 20 s, and a final extension at 72 °C for 5 min. It is highly recommended to optimize the conditions for each new colony PCR primer pair or modified DNA polymerase (*cf*. Figure 3A). The seal was then removed, and the PCR plate was placed in an acoustic dispenser (Echo525 or Echo650T) to dispense 100 nL of the barcode primer combinations into each well. Three microliters of amplification master mix containing Q5 DNA polymerase, buffer, dNTPs, and water was then added to each well for a total reaction volume of 5 μL (four-channel Cobra, ARI). The PCR plate was sealed, and the PCR reactions were performed with the following settings: 98 °C for 30 s, 30 cycles of 98 °C, 20 s; 98 °C, 20 s; and 66 °C, 20 s, and a final extension at 72 °C for 5 min. Reactions were pooled and purified using an open source magnetic bead method prior to the preparation of the Nanopore sequencing library (see above).

### Enzymatic Fragmentation

Enzyme purification and storage were performed according to Hennig et al.^22^ In this study, the transposase Tn5_R27S,E54K,L372P_ was used throughout, as it was reported to generate fragments larger than those of regular Tn5. The respective plasmids can be requested from the authors of Henning et al. Fragmentation reactions were performed as described in Vonesch et al.^23^ Tn5_R27S,E54K,L372P_ (0.5 mg/mL) was loaded with annealed Tn5ME-M13 adaptors (SLo0100 annealed to SLo0673 and SLo0673, respectively) and used for fragmentation of 10 ng of plasmid DNA in 5 μL at 55 °C for 30 s followed by Tn5 inactivation for 5 min at 80 °C; the reaction was performed in a PCR cycler. Then, 2 μL of fragmented DNA was used as a template for barcode PCR using Kapa HiFi (Roche) in 7 μL reactions with the following program: 72 °C, 3 min (critical step for barcoding procedure); 95 °C, 30 s; 20 cycles of 98 °C, 20 s; 66 °C, 15 s; and 72 °C, 5 min, with a final extension at 72 °C for 10 min. Reactions were pooled and purified prior to Nanopore sequencing library preparation. Of note, in-house purified transposase stored at −70 °C has been used for >18 months with no observed loss of activity.

### Library Preparation, Sequencing, and Data Analysis Pipeline

One to two micrograms of barcoded DNA was used as input for library preparation using the SQK-LSK109 kit. Library preparation was performed according to the manufacturer’s guidelines. Each library was sequenced on a single Flongle Flow Cell (FLO-FLG001 [R9.4.1]). Basecalling of raw sequencing data was performed using guppy (up to version 6.5.7; Oxford Nanopore Technologies). The basecalled data were passed to the described DuBA.flow analysis pipeline (https://github.com/RGSchindler/DuBA.flow) together with the necessary sample information and data for analysis. The DuBA.flow pipeline is available as a Docker image from Docker for ease of use and to avoid version incompatibilities; detailed documentation is maintained on GitHub and should be followed. In general, DuBA.flow uses MiniBar (version 0.25)^13^ for demultiplexing, minimap2 (version 2.26-r1175)^14^ for reference mapping, and Samtools (version 1.17)^15^ and Chopper (version 0.5.0)^24^ for quality control. DeepTools (version 3.5.2)^16^ is used to visualize the resulting report file. The report file is returned in .html format and is compatible with all tested browsers (e.g., Mozilla Firefox and Google Chrome). Example data sets are provided within the computational analysis pipeline documentation on GitHub, and example report files are provided with the supporting data (Supporting Data S1).

### Automated Reference Creator Based on Sequencing Reads

The ref.creator pipeline is available as a Docker image from Docker Hub for ease of use and to avoid version incompatibilities; detailed documentation is maintained on GitHub and should be followed (https://github.com/RGSchindler/Ref.creator/). Briefly, ref.creator uses Chopper (version 0.5.0)^24^ to filter and trim reads prior to demultiplexing by MiniBar (version 0.25).^13^ Minimap2 (version 2.26-r1175)^14^ is used for self-alignment and miniasm^25^ to generate the *de novo* assembly. Minipolish^26^ is used to polish the assembly, resulting in the {sample_id}.fasta reference file. To obtain a reference, coverage must be >5-fold or ref.creator may fail to generate a *de novo* assembly. In some cases, multiple contigs will be obtained, e.g., no single plasmid is sequenced or the sample contains a contaminant. However, DuBA.flow is only compatible with a single contig, so the user must reduce the fasta file to a single contig if necessary.

## Data Availability

All materials and data are available in the manuscript and its Supporting Information, the GitHub repository and Docker Hub, or from the corresponding author upon request; transposase material is from Hennig et al. and must be requested from the appropriate source.

## Supporting Information Available

Supporting Information: Table for barcode containing primer (PDF)
Supporting Data S1: HTML example sequencing reports (ZIP)
