# Supplementary material for: DuBA.flow—A Low-Cost, Long-Read Amplicon Sequencing Workflow for the Validation of Synthetic DNA Constructs
Source: ACS Synth Biol. 2024 Jan 31;13(2):457–65. doi: 10.1021/acssynbio.3c00522 (PMC10877597; doi:10.1021/acssynbio.3c00522)
Supplement: Supplementary file 1 — sb3c00522_si_001.pdf [file sb3c00522_si_001.pdf]

Supporting Information

Title: DuBA.flow – A low-cost, long-read amplicon sequencing workflow for the validation of synthetic DNA constructs

Authors: Adán A. Ramírez Rojas[1], Cedric K. Brinkmann[1], Tania S. Köbel[1], Daniel Schindler[1,2]

[1] Max Planck Institute for Terrestrial Microbiology, Karl-von-Frisch-Str. 10, 35043 Marburg, Germany

[2] Center for Synthetic Microbiology, Philipps-University Marburg, Karl-von-Frisch-Str. 14, 35032 Marburg, Germany

This file contains:

Table S1

Tab. S1 | Barcoding primer sequences used in this study. M13 forward and reverse sequences are indicated bold.

Forward barcoding primer		Reverse barcoding primer	
**ID**	**Sequence (5´->3´)**	**ID**	**Sequence (5´->3´)**
SLo2420	AAGAAAGTTGTCGGTGTCTTTGTG**CCCAGTCACGACGTTGTAAAACG**	SLo2516	AAGAAAGTTGTCGGTGTCTTTGTG**AGCGGATAACAATTTCACACAGG**
SLo2421	TCGATTCCGTTTGTAGTCGTCTGT**CCCAGTCACGACGTTGTAAAACG**	SLo2517	TCGATTCCGTTTGTAGTCGTCTGT**AGCGGATAACAATTTCACACAGG**
SLo2422	GAGTCTTGTGTCCCAGTTACCAGG**CCCAGTCACGACGTTGTAAAACG**	SLo2518	GAGTCTTGTGTCCCAGTTACCAGG**AGCGGATAACAATTTCACACAGG**
SLo2423	TTCGGATTCTATCGTGTTTCCCTA**CCCAGTCACGACGTTGTAAAACG**	SLo2519	TTCGGATTCTATCGTGTTTCCCTA**AGCGGATAACAATTTCACACAGG**
SLo2424	CTTGTCCAGGGTTTGTGTAACCTT**CCCAGTCACGACGTTGTAAAACG**	SLo2520	CTTGTCCAGGGTTTGTGTAACCTT**AGCGGATAACAATTTCACACAGG**
SLo2425	TTCTCGCAAAGGCAGAAAGTAGTC**CCCAGTCACGACGTTGTAAAACG**	SLo2521	TTCTCGCAAAGGCAGAAAGTAGTC**AGCGGATAACAATTTCACACAGG**
SLo2426	GTGTTACCGTGGGAATGAATCCTT**CCCAGTCACGACGTTGTAAAACG**	SLo2522	GTGTTACCGTGGGAATGAATCCTT**AGCGGATAACAATTTCACACAGG**
SLo2427	TTCAGGGAACAAACCAAGTTACGT**CCCAGTCACGACGTTGTAAAACG**	SLo2523	TTCAGGGAACAAACCAAGTTACGT**AGCGGATAACAATTTCACACAGG**
SLo2428	AACTAGGCACAGCGAGTCTTGGTT**CCCAGTCACGACGTTGTAAAACG**	SLo2524	AACTAGGCACAGCGAGTCTTGGTT**AGCGGATAACAATTTCACACAGG**
SLo2429	AAGCGTTGAAACCTTTGTCCTCTC**CCCAGTCACGACGTTGTAAAACG**	SLo2525	AAGCGTTGAAACCTTTGTCCTCTC**AGCGGATAACAATTTCACACAGG**
SLo2430	GTTTCATCTATCGGAGGGAATGGA**CCCAGTCACGACGTTGTAAAACG**	SLo2526	GTTTCATCTATCGGAGGGAATGGA**AGCGGATAACAATTTCACACAGG**
SLo2431	CAGGTAGAAAGAAGCAGAATCGGA**CCCAGTCACGACGTTGTAAAACG**	SLo2527	CAGGTAGAAAGAAGCAGAATCGGA**AGCGGATAACAATTTCACACAGG**
SLo2432	AGAACGACTTCCATACTCGTGTGA**CCCAGTCACGACGTTGTAAAACG**	SLo2528	AGAACGACTTCCATACTCGTGTGA**AGCGGATAACAATTTCACACAGG**
SLo2433	AACGAGTCTCTTGGGACCCATAGA**CCCAGTCACGACGTTGTAAAACG**	SLo2529	AACGAGTCTCTTGGGACCCATAGA**AGCGGATAACAATTTCACACAGG**
SLo2434	AGGTCTACCTCGCTAACACCACTG**CCCAGTCACGACGTTGTAAAACG**	SLo2530	AGGTCTACCTCGCTAACACCACTG**AGCGGATAACAATTTCACACAGG**
SLo2435	CGTCAACTGACAGTGGTTCGTACT**CCCAGTCACGACGTTGTAAAACG**	SLo2531	CGTCAACTGACAGTGGTTCGTACT**AGCGGATAACAATTTCACACAGG**
SLo2436	ACCCTCCAGGAAAGTACCTCTGAT**CCCAGTCACGACGTTGTAAAACG**	SLo2532	ACCCTCCAGGAAAGTACCTCTGAT**AGCGGATAACAATTTCACACAGG**
SLo2437	CCAAACCCAACAACCTAGATAGGC**CCCAGTCACGACGTTGTAAAACG**	SLo2533	CCAAACCCAACAACCTAGATAGGC**AGCGGATAACAATTTCACACAGG**
SLo2438	GTTCCTCGTGCAGTGTCAAGAGAT**CCCAGTCACGACGTTGTAAAACG**	SLo2534	GTTCCTCGTGCAGTGTCAAGAGAT**AGCGGATAACAATTTCACACAGG**
SLo2439	TTGCGTCCTGTTACGAGAACTCAT**CCCAGTCACGACGTTGTAAAACG**	SLo2535	TTGCGTCCTGTTACGAGAACTCAT**AGCGGATAACAATTTCACACAGG**
SLo2440	GAGCCTCTCATTGTCCGTTCTCTA**CCCAGTCACGACGTTGTAAAACG**	SLo2536	GAGCCTCTCATTGTCCGTTCTCTA**AGCGGATAACAATTTCACACAGG**
SLo2441	ACCACTGCCATGTATCAAAGTACG**CCCAGTCACGACGTTGTAAAACG**	SLo2537	ACCACTGCCATGTATCAAAGTACG**AGCGGATAACAATTTCACACAGG**
SLo2442	CTTACTACCCAGTGAACCTCCTCG**CCCAGTCACGACGTTGTAAAACG**	SLo2538	CTTACTACCCAGTGAACCTCCTCG**AGCGGATAACAATTTCACACAGG**
SLo2443	GCATAGTTCTGCATGATGGGTTAG**CCCAGTCACGACGTTGTAAAACG**	SLo2539	GCATAGTTCTGCATGATGGGTTAG**AGCGGATAACAATTTCACACAGG**
SLo2444	GTAAGTTGGGTATGCAACGCAATG**CCCAGTCACGACGTTGTAAAACG**	SLo2540	GTAAGTTGGGTATGCAACGCAATG**AGCGGATAACAATTTCACACAGG**
SLo2445	CATACAGCGACTACGCATTCTCAT**CCCAGTCACGACGTTGTAAAACG**	SLo2541	CATACAGCGACTACGCATTCTCAT**AGCGGATAACAATTTCACACAGG**
SLo2446	CGACGGTTAGATTCACCTCTTACA**CCCAGTCACGACGTTGTAAAACG**	SLo2542	CGACGGTTAGATTCACCTCTTACA**AGCGGATAACAATTTCACACAGG**
SLo2447	TGAAACCTAAGAAGGCACCGTATC**CCCAGTCACGACGTTGTAAAACG**	SLo2543	TGAAACCTAAGAAGGCACCGTATC**AGCGGATAACAATTTCACACAGG**

Forward barcoding primer		Reverse barcoding primer	
**ID**	**Sequence (5´->3´)**	**ID**	**Sequence (5´->3´)**
SLo2448	CTAGACACCTTGGGTTGACAGACC**CCCAGTCACGACGTTGTAAAACG**	SLo2544	CTAGACACCTTGGGTTGACAGACC**AGCGGATAACAATTTCACACAGG**
SLo2449	TCAGTGAGGATCTACTTCGACCCA**CCCAGTCACGACGTTGTAAAACG**	SLo2545	TCAGTGAGGATCTACTTCGACCCA**AGCGGATAACAATTTCACACAGG**
SLo2450	TGCGTACAGCAATCAGTTACATTG**CCCAGTCACGACGTTGTAAAACG**	SLo2546	TGCGTACAGCAATCAGTTACATTG**AGCGGATAACAATTTCACACAGG**
SLo2451	CCAGTAGAAGTCCGACAACGTCAT**CCCAGTCACGACGTTGTAAAACG**	SLo2547	CCAGTAGAAGTCCGACAACGTCAT**AGCGGATAACAATTTCACACAGG**
SLo2452	CAGACTTGGTACGGTTGGGTAACT**CCCAGTCACGACGTTGTAAAACG**	SLo2548	CAGACTTGGTACGGTTGGGTAACT**AGCGGATAACAATTTCACACAGG**
SLo2453	GGACGAAGAACTCAAGTCAAAGGC**CCCAGTCACGACGTTGTAAAACG**	SLo2549	GGACGAAGAACTCAAGTCAAAGGC**AGCGGATAACAATTTCACACAGG**
SLo2454	CTACTTACGAAGCTGAGGGACTGC**CCCAGTCACGACGTTGTAAAACG**	SLo2550	CTACTTACGAAGCTGAGGGACTGC**AGCGGATAACAATTTCACACAGG**
SLo2455	ATGTCCCAGTTAGAGGAGGAAACA**CCCAGTCACGACGTTGTAAAACG**	SLo2551	ATGTCCCAGTTAGAGGAGGAAACA**AGCGGATAACAATTTCACACAGG**
SLo2456	GCTTGCGATTGATGCTTAGTATCA**CCCAGTCACGACGTTGTAAAACG**	SLo2552	GCTTGCGATTGATGCTTAGTATCA**AGCGGATAACAATTTCACACAGG**
SLo2457	ACCACAGGAGGACGATACAGAGAA**CCCAGTCACGACGTTGTAAAACG**	SLo2553	ACCACAGGAGGACGATACAGAGAA**AGCGGATAACAATTTCACACAGG**
SLo2458	CCACAGTGTCAACTAGAGCCTCTC**CCCAGTCACGACGTTGTAAAACG**	SLo2554	CCACAGTGTCAACTAGAGCCTCTC**AGCGGATAACAATTTCACACAGG**
SLo2459	TAGTTTGGATGACCAAGGATAGCC**CCCAGTCACGACGTTGTAAAACG**	SLo2555	TAGTTTGGATGACCAAGGATAGCC**AGCGGATAACAATTTCACACAGG**
SLo2460	GGAGTTCGTCCAGAGAAGTACACG**CCCAGTCACGACGTTGTAAAACG**	SLo2556	GGAGTTCGTCCAGAGAAGTACACG**AGCGGATAACAATTTCACACAGG**
SLo2461	CTACGTGTAAGGCATACCTGCCAG**CCCAGTCACGACGTTGTAAAACG**	SLo2557	CTACGTGTAAGGCATACCTGCCAG**AGCGGATAACAATTTCACACAGG**
SLo2462	CTTTCGTTGTTGACTCGACGGTAG**CCCAGTCACGACGTTGTAAAACG**	SLo2558	CTTTCGTTGTTGACTCGACGGTAG**AGCGGATAACAATTTCACACAGG**
SLo2463	AGTAGAAAGGGTTCCTTCCCACTC**CCCAGTCACGACGTTGTAAAACG**	SLo2559	AGTAGAAAGGGTTCCTTCCCACTC**AGCGGATAACAATTTCACACAGG**
SLo2464	GATCCAACAGAGATGCCTTCAGTG**CCCAGTCACGACGTTGTAAAACG**	SLo2560	GATCCAACAGAGATGCCTTCAGTG**AGCGGATAACAATTTCACACAGG**
SLo2465	GCTGTGTTCCACTTCATTCTCCTG**CCCAGTCACGACGTTGTAAAACG**	SLo2561	GCTGTGTTCCACTTCATTCTCCTG**AGCGGATAACAATTTCACACAGG**
SLo2466	GTGCAACTTTCCCACAGGTAGTTC**CCCAGTCACGACGTTGTAAAACG**	SLo2562	GTGCAACTTTCCCACAGGTAGTTC**AGCGGATAACAATTTCACACAGG**
SLo2467	CATCTGGAACGTGGTACACCTGTA**CCCAGTCACGACGTTGTAAAACG**	SLo2563	CATCTGGAACGTGGTACACCTGTA**AGCGGATAACAATTTCACACAGG**
SLo2468	ACTGGTGCAGCTTTGAACATCTAG**CCCAGTCACGACGTTGTAAAACG**	SLo2564	ACTGGTGCAGCTTTGAACATCTAG**AGCGGATAACAATTTCACACAGG**
SLo2469	ATGGACTTTGGTAACTTCCTGCGT**CCCAGTCACGACGTTGTAAAACG**	SLo2565	ATGGACTTTGGTAACTTCCTGCGT**AGCGGATAACAATTTCACACAGG**
SLo2470	GTTGAATGAGCCTACTGGGTCCTC**CCCAGTCACGACGTTGTAAAACG**	SLo2566	GTTGAATGAGCCTACTGGGTCCTC**AGCGGATAACAATTTCACACAGG**
SLo2471	TGAGAGACAAGATTGTTCGTGGAC**CCCAGTCACGACGTTGTAAAACG**	SLo2567	TGAGAGACAAGATTGTTCGTGGAC**AGCGGATAACAATTTCACACAGG**
SLo2472	AGATTCAGACCGTCTCATGCAAAG**CCCAGTCACGACGTTGTAAAACG**	SLo2568	AGATTCAGACCGTCTCATGCAAAG**AGCGGATAACAATTTCACACAGG**
SLo2473	CAAGAGCTTTGACTAAGGAGCATG**CCCAGTCACGACGTTGTAAAACG**	SLo2569	CAAGAGCTTTGACTAAGGAGCATG**AGCGGATAACAATTTCACACAGG**
SLo2474	TGGAAGATGAGACCCTGATCTACG**CCCAGTCACGACGTTGTAAAACG**	SLo2570	TGGAAGATGAGACCCTGATCTACG**AGCGGATAACAATTTCACACAGG**
SLo2475	TCACTACTCAACAGGTGGCATGAA**CCCAGTCACGACGTTGTAAAACG**	SLo2571	TCACTACTCAACAGGTGGCATGAA**AGCGGATAACAATTTCACACAGG**
SLo2476	GCTAGGTCAATCTCCTTCGGAAGT**CCCAGTCACGACGTTGTAAAACG**	SLo2572	GCTAGGTCAATCTCCTTCGGAAGT**AGCGGATAACAATTTCACACAGG**

Forward barcoding primer		Reverse barcoding primer	
**ID**	**Sequence (5´->3´)**	**ID**	**Sequence (5´->3´)**
SLo2477	CAGGTTACTCCTCCGTGAGTCTGA**CCCAGTCACGACGTTGTAAAACG**	SLo2573	CAGGTTACTCCTCCGTGAGTCTGA**AGCGGATAACAATTTCACACAGG**
SLo2478	TCAATCAAGAAGGGAAAGCAAGGT**CCCAGTCACGACGTTGTAAAACG**	SLo2574	TCAATCAAGAAGGGAAAGCAAGGT**AGCGGATAACAATTTCACACAGG**
SLo2479	CATGTTCAACCAAGGCTTCTATGG**CCCAGTCACGACGTTGTAAAACG**	SLo2575	CATGTTCAACCAAGGCTTCTATGG**AGCGGATAACAATTTCACACAGG**
SLo2480	AGAGGGTACTATGTGCCTCAGCAC**CCCAGTCACGACGTTGTAAAACG**	SLo2576	AGAGGGTACTATGTGCCTCAGCAC**AGCGGATAACAATTTCACACAGG**
SLo2481	CACCCACACTTACTTCAGGACGTA**CCCAGTCACGACGTTGTAAAACG**	SLo2577	CACCCACACTTACTTCAGGACGTA**AGCGGATAACAATTTCACACAGG**
SLo2482	TTCTGAAGTTCCTGGGTCTTGAAC**CCCAGTCACGACGTTGTAAAACG**	SLo2578	TTCTGAAGTTCCTGGGTCTTGAAC**AGCGGATAACAATTTCACACAGG**
SLo2483	GACAGACACCGTTCATCGACTTTC**CCCAGTCACGACGTTGTAAAACG**	SLo2579	GACAGACACCGTTCATCGACTTTC**AGCGGATAACAATTTCACACAGG**
SLo2484	TTCTCAGTCTTCCTCCAGACAAGG**CCCAGTCACGACGTTGTAAAACG**	SLo2580	TTCTCAGTCTTCCTCCAGACAAGG**AGCGGATAACAATTTCACACAGG**
SLo2485	CCGATCCTTGTGGCTTCTAACTTC**CCCAGTCACGACGTTGTAAAACG**	SLo2581	CCGATCCTTGTGGCTTCTAACTTC**AGCGGATAACAATTTCACACAGG**
SLo2486	GTTTGTCATACTCGTGTGCTCACC**CCCAGTCACGACGTTGTAAAACG**	SLo2582	GTTTGTCATACTCGTGTGCTCACC**AGCGGATAACAATTTCACACAGG**
SLo2487	GAATCTAAGCAAACACGAAGGTGG**CCCAGTCACGACGTTGTAAAACG**	SLo2583	GAATCTAAGCAAACACGAAGGTGG**AGCGGATAACAATTTCACACAGG**
SLo2488	TACAGTCCGAGCCTCATGTGATCT**CCCAGTCACGACGTTGTAAAACG**	SLo2584	TACAGTCCGAGCCTCATGTGATCT**AGCGGATAACAATTTCACACAGG**
SLo2489	ACCGAGATCCTACGAATGGAGTGT**CCCAGTCACGACGTTGTAAAACG**	SLo2585	ACCGAGATCCTACGAATGGAGTGT**AGCGGATAACAATTTCACACAGG**
SLo2490	CCTGGGAGCATCAGGTAGTAACAG**CCCAGTCACGACGTTGTAAAACG**	SLo2586	CCTGGGAGCATCAGGTAGTAACAG**AGCGGATAACAATTTCACACAGG**
SLo2491	TAGCTGACTGTCTTCCATACCGAC**CCCAGTCACGACGTTGTAAAACG**	SLo2587	TAGCTGACTGTCTTCCATACCGAC**AGCGGATAACAATTTCACACAGG**
SLo2492	AAGAAACAGGATGACAGAACCCTC**CCCAGTCACGACGTTGTAAAACG**	SLo2588	AAGAAACAGGATGACAGAACCCTC**AGCGGATAACAATTTCACACAGG**
SLo2493	TACAAGCATCCCAACACTTCCACT**CCCAGTCACGACGTTGTAAAACG**	SLo2589	TACAAGCATCCCAACACTTCCACT**AGCGGATAACAATTTCACACAGG**
SLo2494	GACCATTGTGATGAACCCTGTTGT**CCCAGTCACGACGTTGTAAAACG**	SLo2590	GACCATTGTGATGAACCCTGTTGT**AGCGGATAACAATTTCACACAGG**
SLo2495	ATGCTTGTTACATCAACCCTGGAC**CCCAGTCACGACGTTGTAAAACG**	SLo2591	ATGCTTGTTACATCAACCCTGGAC**AGCGGATAACAATTTCACACAGG**
SLo2496	CGACCTGTTTCTCAGGGATACAAC**CCCAGTCACGACGTTGTAAAACG**	SLo2592	CGACCTGTTTCTCAGGGATACAAC**AGCGGATAACAATTTCACACAGG**
SLo2497	AACAACCGAACCTTTGAATCAGAA**CCCAGTCACGACGTTGTAAAACG**	SLo2593	AACAACCGAACCTTTGAATCAGAA**AGCGGATAACAATTTCACACAGG**
SLo2498	TCTCGGAGATAGTTCTCACTGCTG**CCCAGTCACGACGTTGTAAAACG**	SLo2594	TCTCGGAGATAGTTCTCACTGCTG**AGCGGATAACAATTTCACACAGG**
SLo2499	CGGATGAACATAGGATAGCGATTC**CCCAGTCACGACGTTGTAAAACG**	SLo2595	CGGATGAACATAGGATAGCGATTC**AGCGGATAACAATTTCACACAGG**
SLo2500	CCTCATCTTGTGAAGTTGTTTCGG**CCCAGTCACGACGTTGTAAAACG**	SLo2596	CCTCATCTTGTGAAGTTGTTTCGG**AGCGGATAACAATTTCACACAGG**
SLo2501	ACGGTATGTCGAGTTCCAGGACTA**CCCAGTCACGACGTTGTAAAACG**	SLo2597	ACGGTATGTCGAGTTCCAGGACTA**AGCGGATAACAATTTCACACAGG**
SLo2502	TGGCTTGATCTAGGTAAGGTCGAA**CCCAGTCACGACGTTGTAAAACG**	SLo2598	TGGCTTGATCTAGGTAAGGTCGAA**AGCGGATAACAATTTCACACAGG**
SLo2503	GTAGTGGACCTAGAACCTGTGCCA**CCCAGTCACGACGTTGTAAAACG**	SLo2599	GTAGTGGACCTAGAACCTGTGCCA**AGCGGATAACAATTTCACACAGG**
SLo2504	AACGGAGGAGTTAGTTGGATGATC**CCCAGTCACGACGTTGTAAAACG**	SLo2600	AACGGAGGAGTTAGTTGGATGATC**AGCGGATAACAATTTCACACAGG**
SLo2505	AGGTGATCCCAACAAGCGTAAGTA**CCCAGTCACGACGTTGTAAAACG**	SLo2601	AGGTGATCCCAACAAGCGTAAGTA**AGCGGATAACAATTTCACACAGG**

Forward barcoding primer		Reverse barcoding primer	
**ID**	**Sequence (5´->3´)**	**ID**	**Sequence (5´->3´)**
SLo2506	TACATGCTCCTGTTGTTAGGGAGG**CCCAGTCACGACGTTGTAAAACG**	SLo2602	TACATGCTCCTGTTGTTAGGGAGG**AGCGGATAACAATTTCACACAGG**
SLo2507	TCTTCTACTACCGATCCGAAGCAG**CCCAGTCACGACGTTGTAAAACG**	SLo2603	TCTTCTACTACCGATCCGAAGCAG**AGCGGATAACAATTTCACACAGG**
SLo2508	ACAGCATCAATGTTTGGCTAGTTG**CCCAGTCACGACGTTGTAAAACG**	SLo2604	ACAGCATCAATGTTTGGCTAGTTG**AGCGGATAACAATTTCACACAGG**
SLo2509	GATGTAGAGGGTACGGTTTGAGGC**CCCAGTCACGACGTTGTAAAACG**	SLo2605	GATGTAGAGGGTACGGTTTGAGGC**AGCGGATAACAATTTCACACAGG**
SLo2510	GGCTCCATAGGAACTCACGCTACT**CCCAGTCACGACGTTGTAAAACG**	SLo2606	GGCTCCATAGGAACTCACGCTACT**AGCGGATAACAATTTCACACAGG**
SLo2511	TTGTGAGTGGAAAGATACAGGACC**CCCAGTCACGACGTTGTAAAACG**	SLo2607	TTGTGAGTGGAAAGATACAGGACC**AGCGGATAACAATTTCACACAGG**
SLo2512	AGTTTCCATCACTTCAGACTTGGG**CCCAGTCACGACGTTGTAAAACG**	SLo2608	AGTTTCCATCACTTCAGACTTGGG**AGCGGATAACAATTTCACACAGG**
SLo2513	GATTGTCCTCAAACTGCCACCTAC**CCCAGTCACGACGTTGTAAAACG**	SLo2609	GATTGTCCTCAAACTGCCACCTAC**AGCGGATAACAATTTCACACAGG**
SLo2514	CCTGTCTGGAAGAAGAATGGACTT**CCCAGTCACGACGTTGTAAAACG**	SLo2610	CCTGTCTGGAAGAAGAATGGACTT**AGCGGATAACAATTTCACACAGG**
SLo2515	CTGAACGGTCATAGAGTCCACCAT**CCCAGTCACGACGTTGTAAAACG**	SLo2611	CTGAACGGTCATAGAGTCCACCAT**AGCGGATAACAATTTCACACAGG**
